# Supplementary material for: Smooth Interpolating Curves with Local Control and Monotone Alternating Curvature
Source: Comput Graph Forum. 2022 Oct 6;41(5):25–38. doi: 10.1111/cgf.14600 (PMC9827861; doi:10.1111/cgf.14600)
Supplement: Supplementary file 1 — Supplement Material [file CGF-41-25-s001.zip › Local-Smooth-Interpolating-MonoCurvature/extern/clothoids/submodules/quarticRootsFlocke/docs/api-cpp/program_listing_file_PolynomialRoots-3-Quartic.cc.html]

Program Listing for File PolynomialRoots-3-Quartic.cc — Quartic Roots v1.0.0

### Navigation

- index
- toc
- Quartic Roots »
- Program Listing for File PolynomialRoots-3-Quartic.cc

# Program Listing for File PolynomialRoots-3-Quartic.cc¶

↰ Return to documentation for file (`PolynomialRoots-3-Quartic.cc`)

```
/*--------------------------------------------------------------------------*\
 |                                                                          |
 |  Copyright (C) 2017                                                      |
 |                                                                          |
 |         , __                 , __                                        |
 |        /|/  \               /|/  \                                       |
 |         | __/ _   ,_         | __/ _   ,_                                |
 |         |   \|/  /  |  |   | |   \|/  /  |  |   |                        |
 |         |(__/|__/   |_/ \_/|/|(__/|__/   |_/ \_/|/                       |
 |                           /|                   /|                        |
 |                           \|                   \|                        |
 |                                                                          |
 |      Enrico Bertolazzi                                                   |
 |      Dipartimento di Ingegneria Industriale                              |
 |      Universita` degli Studi di Trento                                   |
 |      email: enrico.bertolazzi@unitn.it                                   |
 |                                                                          |
\*--------------------------------------------------------------------------*/

#ifdef __GNUC__
#pragma GCC diagnostic ignored "-Wunused-function"
#endif
#ifdef __clang__
#pragma clang diagnostic ignored "-Wglobal-constructors"
#pragma clang diagnostic ignored "-Wvla-extension"
#pragma clang diagnostic ignored "-Wvla"
#pragma clang diagnostic ignored "-Wunused-function"
#pragma clang diagnostic ignored "-Wdocumentation-unknown-command"
#endif

#include "PolynomialRoots.hh"

#include <cmath>
#include <iostream>
#include <algorithm>
#include <limits>

#ifndef DOXYGEN_SHOULD_SKIP_THIS

#define MAX_ITER_SAFETY 50

namespace PolynomialRoots {

  using std::abs;
  static valueType const machepsi = std::numeric_limits<valueType>::epsilon();

  indexType
  Quartic::getRealRoots( valueType r[] ) const {
    indexType nr = 0;
    if ( !cplx0() ) r[nr++] = r0;
    if ( !cplx1() ) r[nr++] = r1;
    if ( !cplx2() ) r[nr++] = r2;
    if ( !cplx3() ) r[nr++] = r3;
    return nr;
  }

  indexType
  Quartic::getPositiveRoots( valueType r[] ) const {
    indexType nr = 0;
    if ( !cplx0() && r0 > 0 ) r[nr++] = r0;
    if ( !cplx1() && r1 > 0 ) r[nr++] = r1;
    if ( !cplx2() && r2 > 0 ) r[nr++] = r2;
    if ( !cplx3() && r3 > 0 ) r[nr++] = r3;
    return nr;
  }

  indexType
  Quartic::getNegativeRoots( valueType r[] ) const {
    indexType nr = 0;
    if ( !cplx0() && r0 < 0 ) r[nr++] = r0;
    if ( !cplx1() && r1 < 0 ) r[nr++] = r1;
    if ( !cplx2() && r2 < 0 ) r[nr++] = r2;
    if ( !cplx3() && r3 < 0 ) r[nr++] = r3;
    return nr;
  }

  indexType
  Quartic::getRootsInRange( valueType a, valueType b, valueType r[] ) const {
    indexType nr = 0;
    if ( !cplx0() && r0 >= a && r0 <= b ) r[nr++] = r0;
    if ( !cplx1() && r1 >= a && r1 <= b ) r[nr++] = r1;
    if ( !cplx2() && r2 >= a && r2 <= b ) r[nr++] = r2;
    if ( !cplx3() && r3 >= a && r3 <= b ) r[nr++] = r3;
    return nr;
  }

  indexType
  Quartic::getRootsInOpenRange( valueType a, valueType b, valueType r[] ) const {
    indexType nr = 0;
    if ( !cplx0() && r0 > a && r0 < b ) r[nr++] = r0;
    if ( !cplx1() && r1 > a && r1 < b ) r[nr++] = r1;
    if ( !cplx2() && r2 > a && r2 < b ) r[nr++] = r2;
    if ( !cplx3() && r3 > a && r3 < b ) r[nr++] = r3;
    return nr;
  }

  // - - - - - - - - - - - - - - - - - - - - - - - - - - - - - - - - - - - - - -
  //
  // x^4 + A x^3 + B x^2 + C x + D
  static
  inline
  void
  scaleQuarticMonicPolynomial(
    valueType   A,
    valueType   B,
    valueType   C,
    valueType   D,
    valueType & AS,
    valueType & BS,
    valueType & CS,
    valueType & DS,
    indexType & i_case,
    valueType & scale
  ) {

    valueType a = std::abs(A);
    valueType b = std::sqrt(abs(B));
    valueType c = std::cbrt(abs(C));
    valueType d = std::sqrt(sqrt(abs(D)));

    if ( a < b ) {
      if ( b < c ) {
        if ( c < d ) i_case = 0; // a < b < c < d        --> d MAX
        else         i_case = 1; // a < b < c and d <= c --> c MAX
      } else {
        if ( b < d ) i_case = 0; // a < b and c <= b < d --> d MAX
        else         i_case = 2; // a < b and c <= b and d <= b --> b MAX
      }
    } else {
      if ( a < c ) {
        if ( c < d ) i_case = 0; // b <= a < c < d        --> d MAX
        else         i_case = 1; // b <= a < c and d <= c --> c MAX
      } else {
        if ( a < d ) i_case = 0; // b <= a and c <= a and a < d --> d MAX
        else         i_case = 3; // b <= a and c <= a and d <= a --> a MAX
      }
    }

    switch ( i_case ) {
      case 0:
        scale = d;
        AS    = A/d;
        BS    = (B/d)/d;
        CS    = ((C/d)/d)/d;
        DS    = D > 0 ? 1 : -1;
      break;
      case 1:
        scale = c;
        AS    = A/c;
        BS    = (B/c)/c;
        CS    = C > 0 ? 1 : -1;
        DS    = (((D/c)/c)/c)/c;
      break;
      case 2:
        scale = b;
        AS    = A/b;
        BS    = B > 0 ? 1 : -1;
        CS    = ((C/b)/b)/b;
        DS    = (((D/b)/b)/b)/b;
      break;
      case 3:
        scale = a;
        AS    = A > 0 ? 1 : -1;
        BS    = (B/a)/a;
        CS    = ((C/a)/a)/a;
        DS    = (((D/a)/a)/a)/a;
      break;
    }
  }

  // - - - - - - - - - - - - - - - - - - - - - - - - - - - - - - - - - - - - - -

  static
  inline
  valueType
  evalHexic(
    valueType x,
    valueType q3,
    valueType q2,
    valueType q1,
    valueType q0
  ) {
    valueType t1 = x + q3;
    valueType t2 = x + t1;
    valueType t3 = x + t2;
    t1 = t1 * x + q2;
    t2 = t2 * x + t1;
    t1 = t1 * x + q1;
    valueType Q    = t1 * x + q0; // Q(x)
    valueType dQ   = t2 * x + t1; // Q'(x)
    valueType ddQ  = t3 * x + t2; // Q''(x) / 2
    valueType dddQ = x + t3;      // Q'''(x) / 6
    return  Q * dddQ * dddQ - dQ * ddQ * dddQ + dQ * dQ; // H(x), usually < 0
  }

  static
  inline
  void
  evalHexic(
    valueType   x,
    valueType   q3,
    valueType   q2,
    valueType   q1,
    valueType   q0,
    valueType & p,
    valueType & dp
  ) {
    valueType t1 = x + q3;
    valueType t2 = x + t1;
    valueType t3 = x + t2;
    t1 = t1 * x + q2;
    t2 = t2 * x + t1;
    t1 = t1 * x + q1;
    valueType Q    = t1 * x + q0; // Q(x)
    valueType dQ   = t2 * x + t1; // Q'(x)
    valueType ddQ  = t3 * x + t2; // Q''(x) / 2
    valueType dddQ = x + t3;      // Q'''(x) / 6
    p  = Q * dddQ * dddQ - dQ * ddQ * dddQ + dQ * dQ; // H(x), usually < 0
    dp = 2 * dddQ * (4 * Q - dQ * dddQ - ddQ * ddQ); // H'(x)
  }

  // - - - - - - - - - - - - - - - - - - - - - - - - - - - - - - - - - - - - - -
  //
  // a4*x^4 + a3*x^3 + a2*x^2 + a1*x + a0 = (x-r)*(a4*x^3+b2*x^2+b1*x+b0)
  static
  void
  deflateQuarticPolynomial(
    valueType   a4,
    valueType   a3,
    valueType   a2,
    valueType   a1,
    valueType   a0,
    valueType   r,
    valueType & b2,
    valueType & b1,
    valueType & b0
  ) {
    indexType i_cross  = 0;
    valueType r2       = r*r;
    valueType v_cross  = std::abs(a0);
    valueType v_cross1 = std::abs(a1*r);
    if ( v_cross1 > v_cross ) { v_cross = v_cross1; i_cross = 1; }
    v_cross1 = std::abs(a2*r2);
    if ( v_cross1 > v_cross ) { v_cross = v_cross1; i_cross = 2; }
    v_cross1 = std::abs(a3*r*r2);
    if ( v_cross1 > v_cross ) { v_cross = v_cross1; i_cross = 3; }
    v_cross1 = std::abs(a4*r2*r2);
    if ( v_cross1 > v_cross ) i_cross = 4;
    switch ( i_cross ) {
      case 0: b2 = a3+a4*r; b1 = a2+r*b2; b0 = a1+r*b1;   break;
      case 1: b2 = a3+a4*r; b1 = a2+r*b2; b0 = -a0/r;     break;
      case 2: b2 = a3+a4*r; b0 = -a0/r;   b1 = (b0-a1)/r; break;
      case 3:
      case 4: b0 = -a0/r; b1 = (b0-a1)/r; b2 = (b1-a2)/r; break;
    }
  }

  /*
  ||   _   _               _              ____  _               _   _
  ||  | \ | | _____      _| |_ ___  _ __ | __ )(_)___  ___  ___| |_(_) ___  _ __
  ||  |  \| |/ _ \ \ /\ / / __/ _ \| '_ \|  _ \| / __|/ _ \/ __| __| |/ _ \| '_ \
  ||  | |\  |  __/\ V  V /| || (_) | | | | |_) | \__ \  __/ (__| |_| | (_) | | | |
  ||  |_| \_|\___| \_/\_/  \__\___/|_| |_|____/|_|___/\___|\___|\__|_|\___/|_| |_|
  */

  // - - - - - - - - - - - - - - - - - - - - - - - - - - - - - - - - - - - - - -
  // Translate to C from Polynomial234RootSolvers
  static
  indexType
  zeroQuarticByNewtonBisection(
    valueType  a,
    valueType  b,
    valueType  c,
    valueType  d,
    valueType & x
  ) {

    valueType p, dp;
    evalMonicQuartic( x, a, b, c, d, p, dp );
    valueType t = p; // save p(x) for sign comparison
    x -= p/dp; // 1st improved root

    indexType iter       = 1;
    indexType oscillate  = 0;
    indexType nconverged = 0;
    bool      bisection  = false;
    bool      converged  = false;
    valueType s(0), u(0); // to mute warning
    while ( ! ( nconverged > 1 || bisection ) && iter < MAX_ITER_SAFETY ) {
      ++iter;
      valueType ddp;
      evalMonicQuartic( x, a, b, c, d, p, dp, ddp );
      if ( p*t < 0 ) { // does Newton start oscillating ?
        if ( p < 0 ) {
          ++oscillate; // increment oscillation counter
          s = x;       // save lower bisection bound
        } else {
          u = x; // save upper bisection bound
        }
        t = p; // save current p(x)
      }
      //dp = (p*dp)/(dp*dp-p*ddp); // double zero correction
      dp = (p*dp)/(dp*dp-0.5*p*ddp); // Halley correction
      //dp = p/dp; // Newton correction
      x -= dp; // new Newton root
      bisection = oscillate > 2; // activate bisection
      converged = std::abs(dp) <= (1+std::abs(x)) * machepsi; // Newton convergence indicator
      if ( converged ) ++nconverged; else nconverged = 0;
    }
    if ( bisection || !converged ) {
      t = u - s; // initial bisection interval
      while ( std::abs(t) > (1+std::abs(x)) * machepsi && iter < MAX_ITER_SAFETY ) { // bisection iterates
        ++iter;
        p = evalMonicQuartic( x, a, b, c, d );
        if ( p < 0 ) s = x;
        else         u = x; // keep bracket on root
        t = (u-s)/2; // new bisection interval
        x = s + t;   // new bisection root
      }
    }
    return iter;
  }

  // - - - - - - - - - - - - - - - - - - - - - - - - - - - - - - - - - - - - - -
  // Translate to C from Polynomial234RootSolvers
  static
  indexType
  zeroHexicByNewtonBisection(
    valueType   q3,
    valueType   q2,
    valueType   q1,
    valueType   q0,
    valueType & x
  ) {
    valueType p, dp;
    evalHexic( x, q3, q2, q1, q0, p, dp );
    valueType t = p; // save p(x) for sign comparison
    x -= p/dp; // 1st improved root

    indexType iter      = 1;
    indexType oscillate = 0;
    bool      bisection = false;
    bool      converged = false;
    valueType s(0), u(0); // to mute warning
    while ( ! (converged||bisection) ) {
      ++iter;
      evalHexic( x, q3, q2, q1, q0, p, dp );
      if ( p*t < 0 ) { // does Newton start oscillating ?
        if ( p < 0 ) {
          ++oscillate; // increment oscillation counter
          s = x;       // save lower bisection bound
        } else {
          u = x; // save upper bisection bound
        }
        t = p; // save current p(x)
      }
      dp = p/dp; // Newton correction
      x -= dp; // new Newton root
      bisection = oscillate > 2; // activate bisection
      converged = std::abs(dp) <= std::abs(x) * machepsi; // Newton convergence indicator
    }
    if ( bisection ) {
      t = u - s; // initial bisection interval
      while ( std::abs(t) > std::abs(x) * machepsi ) { // bisection iterates
        ++iter;
        p = evalHexic( x, q3, q2, q1, q0 );
        if ( p < 0 ) s = x;
        else         u = x; // keep bracket on root
        t = (u-s)/2; // new bisection interval
        x = s + t;   // new bisection root
      }
    }
    return iter;
  }

  // - - - - - - - - - - - - - - - - - - - - - - - - - - - - - - - - - - - - - -

  /*
  ||       _       __ _       _
  ||    __| | ___ / _| | __ _| |_ ___
  ||   / _` |/ _ \ |_| |/ _` | __/ _ \
  ||  | (_| |  __/  _| | (_| | ||  __/
  ||   \__,_|\___|_| |_|\__,_|\__\___|
  */
  // - - - - - - - - - - - - - - - - - - - - - - - - - - - - - - - - - - - - - -
  //
  // a3*x^3 + a2*x^2 + a1*x + a0 = (x-r)*(a3*x^2+b1*x+b0)


  /*\
   *  Calculate the zeros of the quartic a*z^4 + b*z^3 + c*z^2 + d*x + e.
  \*/

  void
  Quartic::findRoots() {
    valueType const & A = ABCDE[0];
    valueType const & B = ABCDE[1];
    valueType const & C = ABCDE[2];
    valueType const & D = ABCDE[3];
    valueType const & E = ABCDE[4];

    iter = nreal = ncplx = 0;

    // special cases
    if ( isZero(A) ) {
      Cubic csolve( B, C, D, E );
      nreal = csolve.numRoots();
      switch ( nreal ) {
        case 3: r2 = csolve.real_root2();
        case 2: r1 = csolve.real_root1();
        case 1: r0 = csolve.real_root0();
      }
      if ( csolve.complexRoots() ) { ncplx = 2; nreal -= 2; }
      return;
    }
    if ( isZero(E) ) {
      Cubic csolve( A, B, C, D );
      nreal = csolve.numRoots();
      r0 = r1 = r2 = r3 = 0;
      switch ( nreal ) {
        case 3: r2 = csolve.real_root2();
        case 2: r1 = csolve.real_root1();
        case 1: r0 = csolve.real_root0();
      }
      ++nreal;
      if ( csolve.complexRoots() ) { ncplx = 2; nreal -= 2; }
      if ( nreal == 4 ) { // caso regolare, 4 radici reali maintain order
        if ( r3 < r2 ) std::swap(r2,r3);
        if ( r2 < r1 ) std::swap(r1,r2);
        if ( r1 < r0 ) std::swap(r0,r1);
      }
      return;
    }
    if ( isZero(B) && isZero(D) ) { // biquadratic case
      // A x^4 + C x^2 + E
      Quadratic qsolve( A, C, E ) ;
      valueType x = qsolve.real_root0() ;
      valueType y = qsolve.real_root1() ;
      if ( qsolve.complexRoots() ) {
        // complex conjugate pair biquadratic roots x +/- iy.
        ncplx = 4;
        x /= 2; // re
        y /= 2; // im
        valueType z = hypot(x,y);
        y = std::sqrt(z - x);
        x = std::sqrt(z + x);
        r0 = -x;
        r1 = y;
        r2 = x;
        r3 = y;
      } else {
        // real roots of quadratic are ordered x <= y
        if ( x >= 0 ) { // y >= 0
          nreal = 4;
          x = std::sqrt(x); y = std::sqrt(y);
          r0 = -y; r1 = -x; r2 =  x; r3 =  y;
        } else if ( y >= 0 ) { // x < 0 && y >= 0
          nreal = ncplx = 2;
          x = std::sqrt(-x); y = std::sqrt(y);
          r0 =  0; r1 = x; // (real,imaginary)
          r2 = -y; r3 = y;
        } else { // x < 0 && y < 0
          ncplx = 4;
          x = std::sqrt(-x); y = std::sqrt(-y);
          r0 = 0; r1 = x; r2 = 0; r3 = y; // 2 x (real,imaginary)
        }
      }
      return;
    }

    /*
    .. The general case. Rescale quartic polynomial, such that largest absolute
    .. coefficient is (exactly!) equal to 1. Honor the presence of a special
    .. quartic case that might have been obtained during the rescaling process
    .. (due to underflow in the coefficients).
    */

    valueType const A3 = B/A;
    valueType const A2 = C/A;
    valueType const A1 = D/A;
    valueType const A0 = E/A;
    valueType q0, q1, q2, q3, scale;
    indexType i_case;
    scaleQuarticMonicPolynomial( A3, A2, A1, A0, q3, q2, q1, q0, i_case, scale );

    // Q(x)    = q0 + q1 * x + q2 * x^2 + q3 * x^3 + x^4
    // Q'(x)/4 = q1/4 + (q2/2) * x + (3/4)*q3 * x^2 + x^3

    /*
    ..  The general quartic case. Search for stationary points. Set the first
    ..  derivative polynomial (cubic) equal to zero and find its roots.
    ..  Check, if any minimum point of Q(x) is below zero, in which case we
    ..  must have real roots for Q(x). Hunt down only the real root, which
    ..  will potentially converge fastest during Newton iterates. The remaining
    ..  roots will be determined by deflation Q(x) -> cubic.
    ..
    ..  The best roots for the Newton iterations are the two on the opposite
    ..  ends, i.e. those closest to the +2 and -2. Which of these two roots
    ..  to take, depends on the location of the Q(x) minima x = s and x = u,
    ..  with s > u. There are three cases:
    ..
    ..     1) both Q(s) and Q(u) < 0
    ..        ----------------------
    ..
    ..        The best root is the one that corresponds to the lowest of
    ..        these minima. If Q(s) is lowest -> start Newton from +2
    ..        downwards (or zero, if s < 0 and a0 > 0). If Q(u) is lowest
    ..        -> start Newton from -2 upwards (or zero, if u > 0 and a0 > 0).
    ..
    ..     2) only Q(s) < 0
    ..        -------------
    ..
    ..        With both sides +2 and -2 possible as a Newton starting point,
    ..        we have to avoid the area in the Q(x) graph, where inflection
    ..        points are present. Solving Q''(x) = 0, leads to solutions
    ..        x = -a3/4 +/- discriminant, i.e. they are centered around -a3/4.
    ..        Since both inflection points must be either on the r.h.s or l.h.s.
    ..        from x = s, a simple test where s is in relation to -a3/4 allows
    ..        us to avoid the inflection point area.
    ..
    ..     3) only Q(u) < 0
    ..        -------------
    ..
    ..        Same of what has been said under 2) but with x = u.
    */

    valueType c2 = 3 * (q3/4);
    valueType c1 = q2/2;
    valueType c0 = q1/4;

    Cubic qsolve( 1, c2, c1, c0 );
    valueType u = qsolve.real_root0(); // root according to paper
    valueType t = qsolve.real_root1();
    valueType s = qsolve.real_root2();

    bool must_refine_r3 = true;
    if ( !qsolve.complexRoots() ) {
      valueType Qs = evalMonicQuartic( s, q3, q2, q1, q0 );
      valueType Qu = evalMonicQuartic( u, q3, q2, q1, q0 );
      bool q0pos = q0 > 0;
      nreal = 1;
      if ( Qs < 0 && Qu < 0 ) {
        if ( Qs < Qu ) {
          r3 = 2;
          if ( q0pos && s < 0 ) r3 = 0;
        } else {
          r3 = -2;
          if ( q0pos && u > 0 ) r3 = 0;
        }
      } else if ( Qs < 0 ) {
        if ( 4*s < -q3 ) {
          r3 = -2;
          if ( q0pos && s > 0 ) r3 = 0;
        } else {
          r3 = 2;
          if ( q0pos && s < 0 ) r3 = 0;
        }
      } else if ( Qu < 0 ) {
        if ( 4*u < -q3 ) {
          r3 = -2;
          if ( q0pos && u > 0 ) r3 = 0;
        } else {
          r3 = 2;
          if ( q0pos && u < 0 ) r3 = 0;
        }
      } else {
        // check for astrological combination when s or u are root of the quartic
        /*
        DO NOT WORK
        valueType epsi = 10 * ( 1 + std::abs(q3) +
                                    std::abs(q2) +
                                    std::abs(q1) +
                                    std::abs(q0) ) * machepsi;
        if      ( Qs <= epsi ) r3 = s;
        else if ( Qu <= epsi ) r3 = u;
        else                   nreal = 0;
        */
        if ( isZero(Qs) ) {
          must_refine_r3 = false; r3 = s;
        } else if ( isZero(Qu) ) {
          must_refine_r3 = false; r3 = u;
        } else {
          nreal = 0;
        }
      }
    } else {
      // one single real root (only 1 minimum)
      valueType Qs = evalMonicQuartic( s, q3, q2, q1, q0 );
      if ( Qs <= 0 ) {
        valueType tmp = q0 >= 0 ? 0 : 2;
        r3 = u > 0 ? -tmp : -2;
        nreal = 1;
      }
    }

    /*
    ..  Do all necessary Newton iterations. In case we have more than 2 oscillations,
    ..  exit the Newton iterations and switch to bisection. Note, that from the
    ..  definition of the Newton starting point, we always have Q(x) > 0 and Q'(x)
    ..  starts (-ve/+ve) for the (-2/+2) starting points and (increase/decrease) smoothly
    ..  and staying (< 0 / > 0). In practice, for extremely shallow Q(x) curves near the
    ..  root, the Newton procedure can overshoot slightly due to rounding errors when
    ..  approaching the root. The result are tiny oscillations around the root. If such
    ..  a situation happens, the Newton iterations are abandoned after 3 oscillations
    ..  and further location of the root is done using bisection starting with the
    ..  oscillation brackets.
    */
    if ( nreal > 0 ) {
      if ( must_refine_r3 )
        iter += zeroQuarticByNewtonBisection( q3, q2, q1, q0, r3 );
      r3 *= scale;

      /*
      ..  Find remaining roots -> reduce to cubic. The reduction to a cubic polynomial
      ..  is done using composite deflation to minimize rounding errors. Also, while
      ..  the composite deflation analysis is done on the reduced quartic, the actual
      ..  deflation is being performed on the original quartic again to avoid enhanced
      ..  propagation of root errors.
      */
      deflateQuarticPolynomial( A, B, C, D, E, r3, q2, q1, q0 );

      Cubic csolve( A, q2, q1, q0 );
      r0 = csolve.real_root0();
      r1 = csolve.real_root1();
      r2 = csolve.real_root2();
      if ( csolve.complexRoots() ) {
        nreal = ncplx = 2;
        if ( r2 > r3 ) std::swap( r2, r3 );
      } else {
        nreal = 4;
        if ( r2 > r3 ) std::swap( r2, r3 );
        if ( r1 > r2 ) std::swap( r1, r2 );
        if ( r0 > r1 ) std::swap( r0, r1 );
      }
    } else {
      /*
      .. If no real roots have been found by now, only complex roots are
      .. possible. Find real parts of roots first, followed by imaginary
      .. components.
      */
      s = q3/2;
      t = s * s - q2;
      u = s * t + q1; // value of Q'(-q3/4) at stationary point -q3/4
      bool notZero = (abs(u) >= machepsi); // H(-a3/4) is considered > 0 at stationary point
      bool minimum;
      if ( !isZero(q3) ) {
        s = q1 / q3;
        minimum = q0 > s * s; // H''(-q3/4) > 0 -> minimum
      } else {
        minimum = 4 * q0 > q2 * q2; // H''(-q3/4) > 0 -> minimum
      }

      bool iterate = notZero || (!notZero && minimum);

      valueType a, b, c, d;
      if ( iterate ) {
        valueType x = q3 >= 0 ? 2 : -2; // initial root -> target = smaller mag root
        iter += zeroHexicByNewtonBisection( q3, q2, q1, q0, x );

        a = x*scale;   // 1st real component -> a
        b = -A3/2 - a; // 2nd real component -> b

        x = 4 * a + A3; // Q'''(a)
        valueType y = x + 2*A3;
        y = y * a + 2*A2; // Q'(a)
        y = y * a + A1;
        y /= x;
        if ( y < 0 ) y = 0; // ensure Q'(a) / Q'''(a) >= 0

        x = 4 * b + A3; // Q'''(b)
        valueType z = x + 2*A3;
        z = z * b + 2*A2; // Q'(b)
        z = z * b + A1;
        z /= x;
        if ( z < 0 ) z = 0; // ensure Q'(b) / Q'''(b) >= 0

        c = a * a;              // store a^2 for later
        d = b * b;              // store b^2 for later
        valueType ss = c + y;   // magnitude^2 of (a + iy) root
        valueType tt = d + z;   // magnitude^2 of (b + iz) root

        if ( ss > tt ) {         // minimize imaginary error
          c = std::sqrt(y);           // 1st imaginary component -> c
          d = std::sqrt(A0 / ss - d); // 2nd imaginary component -> d
        } else {
          c = std::sqrt(A0 / tt - c); // 1st imaginary component -> c
          d = std::sqrt(z);           // 2nd imaginary component -> d
        }

      } else { // no bisection -> real components equal

        a = -A3/4; // 1st real component -> a
        b = a;     // 2nd real component -> b = a

        valueType x = a + A3;
        x = x * a + A2; // Q(a)
        x = x * a + A1;
        x = x * a + A0;
        valueType y = A2/2 - 3*(a*a); // Q''(a) / 2
        valueType z = y * y - x;
        z = z > 0 ? std::sqrt(z) : 0;     // force discriminant to be >= 0
                                     // square root of discriminant
        y = y > 0 ? y + z : y - z;   // larger magnitude root
        x /= y;                      // smaller magnitude root
        c = y < 0 ? 0 : std::sqrt(y);     // ensure root of biquadratic > 0
        d = x < 0 ? 0 : std::sqrt(x);     // large magnitude imaginary component
      }

      ncplx = 4;
      if      (a > b) { r0 = a; r1 = c; r2 = b; r3 = d; }
      else if (a < b) { r0 = b; r1 = d; r2 = a; r3 = c; }
      else            { r0 = a; r1 = c; r2 = a; r3 = d; }
    }
  }

  void
  Quartic::info( std::ostream & s ) const {
    valueType const & A = ABCDE[0];
    valueType const & B = ABCDE[1];
    valueType const & C = ABCDE[2];
    valueType const & D = ABCDE[3];
    valueType const & E = ABCDE[4];

    s << "\npoly a=" << A << " b=" << B << " c=" << C << " d=" << D << " e=" << E
      << "\nn. complex = " << ncplx
      << "\nn. real    = " << nreal;
    if ( ncplx > 0 ) {
      s << "\nx0 = (" << r0 << "," <<  r1 << ')'
        << "\nx1 = (" << r0 << "," << -r1 << ')';
    } else {
      if ( nreal > 0 ) s << "\nx0 = " << r0;
      if ( nreal > 1 ) s << "\nx1 = " << r1;
    }
    if ( ncplx > 2 ) {
      s << "\nx2 = (" << r2 << "," <<  r3 << ')'
        << "\nx3 = (" << r2 << "," << -r3 << ')';
    } else {
      if ( nreal > 2 || (ncplx > 0 && nreal > 0) ) s << "\nx2 = " << r2;
      if ( nreal > 3 || (ncplx > 0 && nreal > 1) ) s << "\nx3 = " << r3;
    }
    s << '\n';
  }

  bool
  Quartic::check( std::ostream & s ) const {
    valueType const & A = ABCDE[0];
    valueType const & B = ABCDE[1];
    valueType const & C = ABCDE[2];
    valueType const & D = ABCDE[3];
    valueType const & E = ABCDE[4];
    bool ok = true;
    valueType epsi = 1000 * ( ( std::abs(A) +
                                std::abs(B) +
                                std::abs(C) +
                                std::abs(D) +
                                std::abs(E) )*machepsi) ;
    if ( ncplx > 0 ) {
      valueType z0 = std::abs(eval( root0() ));
      valueType z1 = std::abs(eval( root1() ));
      s << "|p(r0)| = " << z0 << "\n|p(r1)| = " << z1 << '\n';
      ok = ok && std::abs(z0) < epsi && std::abs(z1) < epsi;
    } else {
      if ( nreal > 0 ) {
        valueType z0 = eval( real_root0() );
        s << "p(r0) = " << z0 << '\n';
        ok = ok && std::abs(z0) < epsi;
      }
      if ( nreal > 1 ) {
        valueType z1 = eval( real_root1() );
        s << "p(r1) = " << z1 << '\n';
        ok = ok && std::abs(z1) < epsi;
      }
    }
    if ( ncplx > 2 ) {
      valueType z2 = std::abs(eval( root2() ));
      valueType z3 = std::abs(eval( root3() ));
      s << "|p(r2)| = " << z2 << "\n|p(r3)| = " << z3 << '\n';
      ok = ok && std::abs(z2) < epsi && std::abs(z3) < epsi;
    } else {
      if ( nreal > 2 || (ncplx > 0 && nreal > 0)  ) {
        valueType z2 = eval( real_root2() );
        s << "p(r2) = " << z2 << '\n';
        ok = ok && std::abs(z2) < epsi;
      }
      if ( nreal > 3 || (ncplx > 0 && nreal > 1)  ) {
        valueType z3 = eval( real_root3() );
        s << "p(r3) = " << z3 << '\n';
        ok = ok && std::abs(z3) < epsi;
      }
    }
    return ok;
  }

}

#endif

// EOF: PolynomialRoots-3-Quartic.cc
```

### Quick search

### Table of Contents

- Quartic Roots Solver
- C++ API

«
hide menu

menu
sidebar
»

### Navigation

- index
- toc
- Quartic Roots »
- Program Listing for File PolynomialRoots-3-Quartic.cc

© Copyright 2021, Enrico Bertolazzi.
Created using Sphinx 4.2.0.
